# Effective protective mechanisms of HO-1 in diabetic complications: a narrative review

**DOI:** 10.1038/s41420-024-02205-x

**Published:** 2024-10-10

**Authors:** Jing-jing Zhang, Ping Ni, Yi Song, Man-jun Gao, Xi-ying Guo, Bao-qing Zhao

**Affiliations:** 1 Medicine Research Institute & Hubei Key Laboratory of Diabetes and Angiopathy, Xianning, Hubei China; 2 Schools of Pharmacy and Hubei University of Science and Technology, Xianning, China; 3 Clinical Medicine, Hubei University of Science and Technology, Xianning, China

**Keywords:** Obesity, Apoptosis

## Abstract

Diabetes mellitus is a metabolic disorder with persistent hyperglycemia caused by a variety of underlying factors. Chronic hyperglycemia can lead to diverse serious consequences and diversified complications, which pose a serious threat to patients. Among the major complications are cardiovascular disease, kidney disease, diabetic foot ulcers, diabetic retinopathy, and neurological disorders. Heme oxygenase 1 (HO-1) is a protective enzyme with antioxidant, anti-inflammatory and anti-apoptotic effects, which has been intensively studied and plays an important role in diabetic complications. By inducing the expression and activity of HO-1, it can enhance the antioxidant, anti-inflammatory, and anti-apoptotic capacity of tissues, and thus reduce the degree of damage in diabetic complications. The present study aims to review the relationship between HO-1 and the pathogenesis of diabetes and its complications. HO-1 is involved in the regulation of macrophage polarization and promotes the M1 state (pro-inflammatory) towards to the M2 state (anti-inflammatory). Induction of HO-1 expression in dendritic cells inhibits them maturation and secretion of pro-inflammatory cytokines and promotes regulatory T cell (T_reg_ cell) responses. The induction of HO-1 can reduce the production of reactive oxygen species, thereby reducing oxidative stress and inflammation. Besides, HO-1 also has an important effect in novel programmed cell death such as pyroptosis and ferroptosis, thereby playing a protective role against diabetes. In conclusion, HO-1 plays a significant role in the occurrence and development of diabetic complications and is closely associated with a variety of complications. HO-1 is anticipated to serve as a novel target for addressing diabetic complications, and it holds promise as a potential therapeutic agent for diabetes and its associated complications. We hope to provide inspiration and ideas for future studies in the mechanism and targets of HO-1 through this review.

## Facts

- Diabetic complications are various and the pathological mechanism is complex.
- HO-1 is involved in a variety of pathological mechanisms of diabetic complications, especially inflammation and cell death caused by oxidative stress.
- HO-1 induction also brings negative effects, inducing ferroptosis, CO toxicity.
- Targeting HO-1 may provide new strategies for the prevention and treatment of diabetes.

## Open questions

- What are the therapeutic targets or strategies suitable for the various diabetic complications?
- How does HO-1 interact or cross-regulation in different modes of cell death?
- How to regulate the dual character of HO-1 in ferroptosis?
- How to develop precise strategies for the toxicity of HO-1 targeted therapy?

## Introduction

**Fig. 1:**
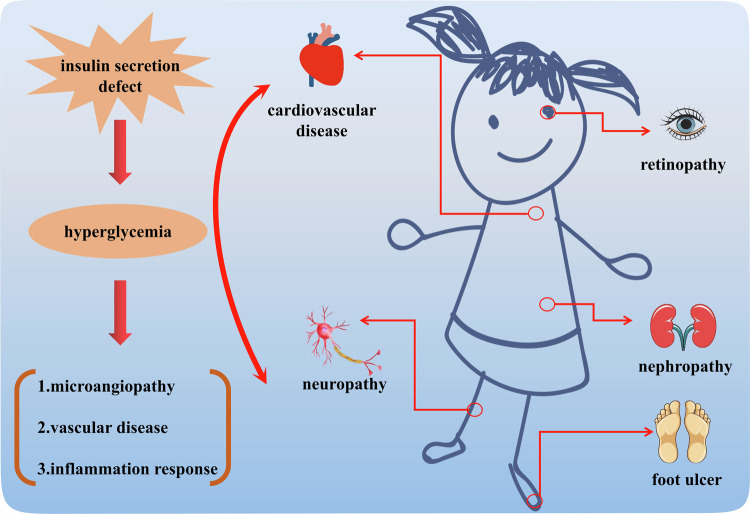
Major complications of diabetes. Defects in insulin secretion caused by a series of problems such as diet will lead to hyperglycemia and further trigger various chronic complications, including cardiovascular disease, retinopathy, nephropathy, neuropathy, foot ulcer.

Diabetes mellitus (DM, abbreviations are defined in Table 1) is a major public health problem worldwide, which imposes heavy economic and medical burdens on patients and healthcare systems [1, 2]. The International Diabetes Federation predicted that the number of individuals inflicted with DM is slated to surpass 578 million globally by 2030, including both developed and developing countries. By 2045, the number of DM patients could soar to a staggering 783.2 million [3, 4]. The development of DM related complications has raised mortality rates and resulted in significant medical expenses. Diabetes patients frequently have a wide range of complications encompassing diabetic cardiovascular diseases, diabetic neuropathy, diabetic foot ulcers, diabetic retinopathy and diabetic nephropathy, which have a substantial negative influence on the physical and psychological health of these individuals (Fig. 1) [5]. Consequently, it is crucial to investigate the underlying causes and effective treatments for diabetes and its associated complications.

**Table 1:** Abbreviations.

Abbreviations			
APCs	antigen-presenting cells	IL	interleukin
BR	bilirubin	MAPK	mitogen-activated protein kinase
BV	biliverdin	MCP-1	monocyte chemotactic protein 1
BVR	biliverdin reductase	MDA	malondialdehyde
CO	carbon monoxides	NETosis	necrotic extracellular traps
DAMPs	danger associated molecular patterns	Nrf2	nuclear factor erythroid 2-related factor2
DFU	diabetic foot ulcer	PAMPs	pathogen associated molecular patterns
DM	diabetes mellitus	ROS	reactive oxygen species
DN	diabetic nephropathy	SDF-1	stromal-derived factor-1
DR	diabetic retinopathy	SOD	superoxide dismutase
EGR-1	early growth response protein 1	TGF-β	transforming growth factor β
GPx4	Glutathione peroxidase 4	TNF-α	tumor necrosis factor α
GSH	glutathione	VDR	vitamin D receptor
HMGB1	high-mobility group box-1	VEGF	vascular endothelial growth factor
HO	heme oxygenase	T_reg_ cells	regulatory T cells

Heme oxygenase (HO) has three distinct isoenzymes, respectively namely heme oxygenase 1 (HO-1), heme oxygenase 2 (HO-2) and heme oxygenase 3 (HO-3) [6]. HO-1 is found in various tissue cells throughout the human body, which has the potential to regulate the onset and advancement of diabetic complications, such as cardiovascular disease, neuropathy, and renal disease [7–9]. HO-1 expression is mainly regulated by the activation of nuclear factor-erythroid2-related factor 2 (Nrf2). In the normal state of cells, Nrf2 binds to kelch-like ECH-associated protein-1 (Keap1) to form a complex, which exists in the cytoplasm. When cells are subjected to oxidative stress, Nrf2 dissociates from Keap1 and moves to the nucleus to bind to the promoter of HO-1, promoting the transcription and expression of HO-1 [10]. Its main function is to catalyze the combination of heme molecules with oxygen, resulting in the formation of oxyhemoglobin. Subsequently, it breaks down oxyhemoglobin into ferric ions, carbon monoxides (CO) and bilirubin (BR) in a process known as heme metabolism. Among them, heme, being essential for all oxygen-demanding organisms, is synthesized from protoporphyrin IX and ferrous ions [11]. Heme produces signaling molecules, such as CO, biliverdin (BV), BR and Fe^2+^, through the catalysis of HO-1. Each of these molecules exerts distinct effects on cellular function, both locally and distally, through individual molecular targets [12, 13]. BV, a breakdown product of HO-1, can potentially enhance the production of BR, a compound with the potential to prevent diabetes. This effect is achieved by increasing HO-1 activity and signaling to biliverdin reductase (BVR). Bilirubin is produced through a reaction catalyzed by both HO-1 and HO-2, which also simultaneously releases CO and Fe^2+^. NADH and NADPH serve as electron donors in this process. The conversion of bilirubin to BRHO-1, catalyzed by BVR-A, plays a role in defending the body against oxidative stress, diseases and injuries [14]. HO-1 and HO-2 are the products of two distinct genes, which are located in chromosome 22 for HO-1 and chromosome 16 for HO-2, respectively. HO-1 and HO-2 degrade heme in the same pattern, releasing biliverdin, CO, and Fe^2+^. In contrast to HO-1, the HO-2 isozyme is constitutively expressed, highly detectable in brain, testis, endothelial and smooth muscle cells from cerebral vessels. Most studies have shown that the presence of HO-2 indicates a crucial role in male reproductive system and brain related diseases, although some studies have found HO-2 in the prevention of kidney injury and diabetes. However, the HO-3 is thought to be a pseudogene processed from HO-2 transcripts, and its function is not known [15, 16].

Research has established an abnormal HO-1 expression among diabetic individuals, especially in those with complications like cardiovascular disease, neuropathy, nephropathy and diabetic foot ulcers [17, 18]. Type 1 diabetes mellitus (T1DM) is a chronic autoimmune disease characterized by impaired insulin secretion, while type 2 diabetes mellitus (T2DM) is mainly caused by insufficient insulin secretion or insulin resistance. HO-1 in β cells and immature dendritic cells can delay autoimmune damage in pancreatic islet transplants and effectively maintain immune tolerance, which helps to delay the onset of T1DM in NOD mice model [19]. In type 2 diabetes, the reduced expression of HO-1 will increase the production of ROS, leading to oxidative stress and inflammatory reaction, further aggravating cell damage (Table 2). The studies mentioned above propose the possibility of the involvement of HO-1 in the control of both diabetes and its related complications.

**Table 2:** Summary of the protective effects against DM by HO-1.

Medicine/reagents	Signal pathway	Mechanisms	Effects	Models	Ref.
Hemin	HO-1/Nrf2/GPx4	Ferroptosis, oxidative stress	Contrast-induced nephropathy	HK-2 cells, SD rats	[171]
Mesenchymal stem cells-derived exosomes	Nrf2/HO-1	Ferroptosis, inflammation, vascular endothelial cells dysfunction	Diabetes with sepsis	HPME cells, HBMS cells	[167]
Ferrostatin-1	Nrf2/HO-1	Ferroptosis	Diabetes-induced liver injury	C57BL/6 mice	[172]
Liraglutide	Nrf2/HO-1/GPx4	Ferroptosis, fibrosis	Diabetes-induced liver injury	HepG2 cells, C57BL/KsJ mice	[168]
Tetramethylpyrazine	SIRT1/Nrf2/ HO-1	Mitochondrial dysfunction and oxidative stress	Diabetic Alzheimer	C57BL/6 mice	[165]
Albiflorin	Nrf2/HO-1/HMGB1/NF-kB	Inflammation, oxidative stress	Diabetic Alzheimer	SD rats	[166]
Soluble epoxide hydrolase	AMPK/HO-1	Oxidative stress	Diabetic blood-brain barrier dysfunction	C57BL/KsJ	[173]
Salidroside	AKT/Nrf2/HO-1	Apoptosis, fibrosis, hypertrophy	Diabetic cardiomyopathy	H9C2cells, C57BL/KsJ mice	[109]
Myricetin	Nrf2/HO-1, IκB-α/NF-κB/p65, TGFβ/Smad3	Inflammation, oxidative stress Inflammation, apoptosis Fibrosis	Diabetic cardiomyopathy	NRCM cells, DCM mice	[110]
6-Gingerol	Nrf2/HO-1	Ferroptosis, inflammation	Diabetic cardiomyopathy	H9C2 cells, C57BL/6 mice	[174]
Curcumin	AKT/Nrf2/ARE	Oxidative stress, pyroptosis		Myocardial cells, SD rats	[73]
	Nrf2/HO-1/GPx4	Ferroptosis	Diabetic cardiomyopathy	Rat H9C2 cells, New Zealand rabbits	[111]
	Nrf2/HO-1	Apoptosis, oxidative stress		H9C2 cells, SD rats	[112]
Sargassum wightii fucoidan	Nrf2/HO-1	Apoptosis, fibrosis, oxidative stress	Diabetic cardiomyopathy	Wistar rats	[175]
Polyherbal formulation	NF-kB/Nrf2/HO-1	Inflammation, oxidative stress	Diabetic cardiomyopathy	Wistar rats	[176]
Piceatannol	Nrf2/HO-1, NF-κB	Apoptosis, fibrosis, inflammation, oxidative stress	Diabetic cardiomyopathy	H9C2 cells, SD rats	[177]
Jasminum sambac phenolics extracted	Nrf2/HO-1	Apoptosis, oxidative stress	Diabetic cardiomyopathy	Wistar albino rats	[178]
Fibroblast growth factor-19	AMPK/Nrf2/HO-1	Oxidative stress	Diabetic cardiomyopathy	Cardiomyocytes, C57BL/6 mice	[179]
Quercetin	Nrf2/HO-1	Apoptosis, fibrosis, pyroptosis	Diabetic cardiomyopathy	H9C2 cells, SD rats	[180]
Sinomenine	EGF/Nrf2/HO-1 via microbiota-gut-brain axis	Ferroptosis	Diabetic cognitive dysfunction	HT-22 cells, SD rats	[181]
Artemisinin	Nrf2/HO-1/GPx4	Ferroptosis	Diabetic cognitive dysfunction	C57BL/6J mice	[182]
Hydrogen sulfide	Nrf2/HO-1/NLRP3	Inflammation, oxidative stress	Diabetic cognitive dysfunction	SD rats	[183]
Soy isoflavones	Nrf2/HO-1	Oxidative stress	Diabetic cognitive dysfunction	GK & Wistar rats	[162]
Apocynin	Nrf2/HO-1	Apoptosis, inflammation, oxidative stress	Diabetic cognitive dysfunction	SD rats	[184]
Betulin	HO-1/Nrf-2/NF-κB	Inflammation, oxidative stress	Diabetic cognitive dysfunction	SD rats	[185]
Sirtuin 1	Nrf2-NF-κB	Inflammation, oxidative stress	Diabetic cognitive dysfunction	SD rats	[163]
JQ-1	Nox4-Nrf2	Apoptosis, inflammation, oxidative stress	Diabetic cognitive dysfunction	PC12 cells, Wistar rats	[164]
Quercetin	KEAP1/Nrf2/HO-1	Ferroptosis	Diabetic encephalopathy	PC12 cells, Goto-Kakizak rats	[170]
Bilirubin	Nrf2/HO-1	Ferroptosis, oxidative stress	Diabetic islet transplantation	BALB/c mice	[170]
Notoginsenoside R1	Nrf2/HO-1/TGF-β	Apoptosis, fibrosis; oxidative stress	Diabetic kidney	HK-2 cells, C57BL/6J mice	[146]
–	ATF4/HO-1	Autophagy, apoptosis	Diabetic kidney	MPC5 cells, C57BL/KsJ mice	[186]
Ferrostatin-1	HIF-1α/HO-1	Ferroptosis	Diabetic kidney	C57BLKs/J mice	[78]
Umbelliferone	Nrf2/HO-1	Ferroptosis	Diabetic kidney	HK-2 cells, C57BL/KsJ mice	[156]
Quercetin	Nrf2/HO-1	Ferroptosis	Diabetic kidney	HK-2 cells, C57BL/KsJ mice	[77]
HMGB1	TLR4/NF-κB/HO-1	Ferroptosis	Diabetic kidney	SV40-MES 13 cells, human serum	[157]
Vitamin D receptor	Nrf2/HO-1	Ferroptosis	Diabetic kidney	HK-2 cells, C57BL/KsJ mice	[82]
Oligo-fucoidan	Nrf2/HO-1	Fibrosis	Diabetic kidney	NRK-52E cells, C57BL/6 mice	[151]
Astaxanthin	Nrf2/ARE	Fibrosis	Diabetic kidney	GMCs cells, SD rats	[149]
KC7F2/hemin	HIF-1α/HO-1	Apoptosis, mitochondrial fragmentation, oxidative stress	Diabetic kidney	HK-2 cells, PEPCK Cre mice	[187]
Baicalin	Nrf2/MAPK	Inflammation, oxidative stress	Diabetic kidney	C57BLKs/J mice	[145]
Telmisartan	Nrf2/HO-1	Apoptosis, angiogenesis, inflammation, oxidative stress	Diabetic kidney	SD rats	[153]
Dapagliflozin	miR-155-5p/HO-1/NLRP3	Pyroptosis	Diabetic kidney	MPC5 cells, C57BL/6 mice	[69]
Syringaresinol	NLRP3/Caspase-1/GSDMD	Pyroptosis	Diabetic kidney	RTE cells, C57BL/6J mice	[72]
Atorvastatin	MALAT1/miR-200c/ Nrf2	Pyroptosis	Diabetic kidney	MPC5 cells	[70]
Triptolide	Nrf2/HO-1/NLRP3	Pyroptosis, oxidative stress	Diabetic kidney	MPC5 cells, C57BL/6J mice	[71]
Shenkang	Keap1/Nrf2/HO-1	Oxidative stress	Diabetic kidney	HK-2 cells, SD rats	[10]
Echinochrome A	AMPKα/Nrf2/HO-1	Fibrosis, mitochondrial function, oxidative stress	Diabetic kidney	C57BL/KsJ mice	[147]
Sinapic acid	Nrf-2/HO-1 Nrf-2/HO-1, NF-kB	Apoptosis, inflammation, oxidative stress	Diabetic kidney Diabetic cardiomyopathy	Wistar rats	[154] [188]
Shenkang Pills	HIF-1α/HO-1	Ferroptosis	Diabetic kidney	C57BL/6 mice	[189]
Fish oil	Nrf2/ARE	Inflammation, oxidative stress	Diabetic cognitive dysfunction	SD rats	[190]
Melatonin	Nrf2/HO-1	Ferroptosis	Diabetic osteoporosis	MC3T3-E1 cells, SD rats	[191]
Maresin-1	Nrf2/HO-1/GPx4	Ferroptosis	Diabetic retinopathy	ARPE-19 cells, C57BL/6 mice	[192]
Amygdalin	Nrf2/ARE	Ferroptosis, oxidative stress	Diabetic retinopathy	HRE cells, SD rats	[161]
n-butylidenephthalide	Nrf2/HO-1	Apoptosis, senescence, tight junction impairment of retinal pigment epithelium	Diabetic retinopathy	ARPE-19 cells, C57BL/6 mice	[193]
Urolithin A	Nrf2/HO-1	Inflammation, oxidative stress	Diabetic retinopathy	HRE cells, SD rats	[194]
Arjunolic acid	AMPK/mTOR/HO-1	Apoptosis, autophagy, inflammation	Diabetic retinopathy	ARPE-19 cells, SD rats	[195]
Hydroxysafflor yellow A	Nrf2/HO-1	Apoptosis	Diabetic retinopathy	Wistar rats	[160]
Platycodin D	TLR4/MyD88/NF-κB Nrf2/HO-1	Inflammation, oxidative stress	Diabetic retinopathy	ARPE-19 cells, SD rats	[196]
Astaxanthin	Nrf2/keap1	Apoptosis, inflammation, oxidative stress	Diabetic retinopathy	SD rats	[197]
Tricin	Sestrin2/Nrf2	Oxidative stress and angiogenesis	Diabetic retinopathy	ARPE-19 cells, SD rats	[198]
Carnosol	ERK/Nrf2/HO-1	Apoptosis, oxidative stress	Diabetic retinopathy	HRE cells	[199]
Rhaponticin	Nrf2/HO-1/NF-κB	Inflammation, oxidative stress	Diabetic retinopathy	Wistar rats	[200]
Nicotinamide mononucleotide	SIRT1/Nrf2/HO-1	Apoptosis, cell migration, and junctions	Diabetic retinopathy	HCECs cells	[201]
sp^2^-Iminosugar Glycolipid Sulfoxide	HO-1/IL-10	Inflammation, M1→M2	Diabetic Retinopathy	Bv.2 cells, Bio-Breeding rats	[159]
MG132	Nrf2/ARE	Oxidative stress, 26s proteasome activity	Diabetic Retinopathy	HCECs cells, SD rats	[202]
Acteoside	Keap1/Nrf2/ARE	Apoptosis, oxidative stress	Diabetic Retinopathy	ARPE-19 cells, C57BL/6 mice	[203]
Scoparia dulcis L. extract	Nrf2/HO-1	Inflammation, oxidative stress	Diabetic Retinopathy	ARPE-19 cells	[204]
Diosgenin	AMPK/Nrf2/HO-1	Inflammation, oxidative stress	Diabetic Retinopathy	ARPE‐19 cells	[205]
Acidic polysaccharides	Nrf2/ARE	Oxidative stress, inhibit angiogenesis	Diabetic Retinopathy	HUVECs cells, C57BL/KsJ mice	[206]
Gynura divaricata (L.) DC.	Nrf2/HO-1	Apoptosis, angiogenesis, oxidative stress	Diabetic wound healing	HUVECs cells, SD rats	[133]
Thermosensitive Hydrogel Incorporating Prussian Blue Nanoparticles	Nrf2/HO-1	Inflammation, oxidative stress	Diabetic wound healing	HUVECs cells, C57BL/6J mice	[207]
gallocatechin (GC)/silver nanoparticles	Nrf2/HO-1	Inflammation, oxidative stress	Diabetic wound healing	SD rats	[208]
San Huang Xiao Yan	AMPK/Nrf2/HMGB1	Inflammation, oxidative stress	Diabetic wound healing	RAW264.7 cells, SD rats	[209]
Circulating exosomal miR-181b-5p	Nrf2/HO-1	Angiogenesis, cells senescence	Diabetic wound healing	HUVECs cells, C57BL/6J mice	[210]
Tetrahedral framework nucleic acids	Akt/Nrf2/HO-1	Inflammation, oxidative stress	Diabetic wound healing	HUVECs cells, Wistar rats	[211]
Liraglutide	HIF-1α/HO-1	Apoptosis, oxidative stress	Diabetic wound healing	HUVECs cells, C57BL/KsJ mice	[134]
Berberine-modified ZnO nano-colloids hydrogel	Nrf2/HO-1/NQO1	Oxidative stress	Diabetic wound healing	HaCaT cells, NIH-3T3 cells, SD rats	[212]
Polygonatum kingianum	Nrf2/HO-1	Inflammation, oxidative stress	Diabetic wound healing	SD rats	[213]
sodium bisulfide	HO-1/TNF-α	Inflammation, oxidative stress	Diabetic wound healing	SD rats	[214]
dimethyl fumarate	Nrf2/HO-1	Inflammation, oxidative stress	Diabetic wound healing	Macrophages, Wistar rats	[215]
Turmeric-derived nanoparticles functionalized aerogel	Nrf2/HO-1	Apoptosis, macrophage polarization, oxidative stress	Diabetic wound healing	L929 cells, RAW264.7 cells, C57BL/6J mice	[40]

## The function of HO-1 in inflammatory reaction

**Fig. 2:**
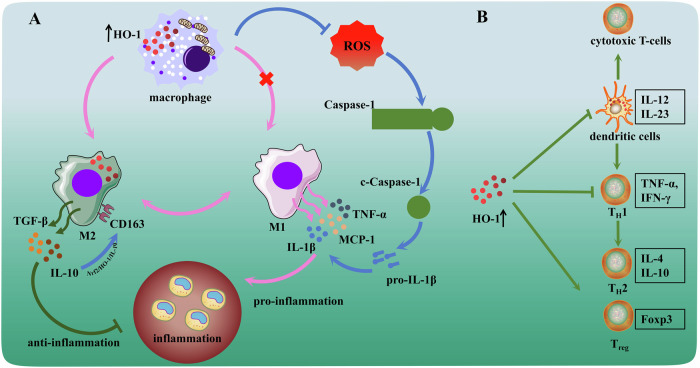
HO-1 reduced inflammatory response via affecting immune regulation. **A** Induction of HO-1 facilitated the differentiation of macrophages into anti-inflammatory M1 and the release of IL-10. And inhibitedthe production of reactive oxygen species to reduce inflammation. **B** HO-1 expression in dendritic cells promotedtheir anti-inflammatory activity and inhibited differentiation of cytotoxic T cells. Induced expression of HO-1 proteindecreases the expression of pro-inflammatory T_H_1 cytokines shifted to a beneficial T_H_2 pattern and promotesantigen-specific T_reg_ cell differentiation.

In various experimental models of inflammation, HO-1 induction produces favorable effects. Inflammatory pathways have been implicated as potential pathogenic mediators of many diseases, including obesity, immune diseases, metabolic diseases and infections [20–22]. Indeed, inflammation has emerged as a crucial determining factor of the evolution of DM, as well as major complications and cardiovascular disease [23, 24]. The human inflammatory response is an intricate biological process that entails the interaction of various immune cells and molecules. The persistence of hyperglycemia in diabetes triggers a sequence of local metabolic, vascular, and neurochemical modifications, which ultimately culminate in vascular disease and microangiopathy [25]. In this process, a vital role in causing considerable harm to the Schwann cells and neurons in the peripheral nervous system is played by pro-inflammatory cytokines produced by cells that are resident or have infiltrated [26]. Macrophages regulate the inflammatory process depending on their differentiation status. Classical macrophages (M1) are responsible for triggering the inflammatory response via the secretion of pro-inflammatory cytokines and reactive oxygen species (ROS). Conversely, alternatively activated macrophages (M2) work towards inflammation resolution and promote tissue remodeling via the discharge of interleukin-10 (IL-10) and transforming growth factor β (TGF-β) [27, 28]. For instance, in a basic inflammatory response like infected tissues, macrophages typically undergo a sequential transition between the M1 and M2 phases. M1 macrophages play a key role in the initial stages of the inflammatory response by releasing considerable amounts of inflammatory agents such as tumor necrosis factor α (TNF-α), IL-6 and IL-12, which contribute to aggravating inflammation. Subsequently, M2 phenotype is polarized from macrophages and primarily responsible for tissue repair and anti-inflammatory pathways via producing mediators such as IL-10 and TGF-β [29]. However, the response is distinct in chronic inflammatory diseases such as atherosclerosis in diabetic mice and diabetic nephropathy [30, 31]. In these disorders, the coexistence of M1 and M2 macrophages results in the formation of complex cellular networks, which exacerbate persistent inflammation and fibrosis, further accelerating disease progression. Studies have demonstrated that induction of HO-1 expression in macrophages yields amplified antioxidant efficacy and mitigates the inflammatory aspect of atherosclerotic lesions. The lowered or nonexistent HO-1 expression within macrophages is linked to an increase in pro-inflammatory cytokine expression, namely monocyte chemotactic protein 1 (MCP-1) and IL-6. Furthermore, this association is also observed in scavenger receptor A expression, leading to the formation of foam cells [32–34]. Interestingly, polarization of M2 macrophages is also associated with HO-1 expression in diabetes [35, 36]. M2 macrophage polarization reduces the apoptosis of interstitial cells of Cajal in diabetic rats, which is mediated by the activation of Nrf2/HO-1 pathway [37]. At the same time, the activation of HO-1-related pathways promotes the polarization of M2 macrophages to improve diabetic-inflammatory response, oxidative stress, and cell proliferation have been verified by various of studies [38–40]. Although studies on the relationship between HO-1 and macrophages in diabetes or its complications are limited, the mechanisms by which macrophages maintain the microenvironment stability of the body and HO-1’s excellent antioxidant and anti-inflammatory properties suggest that targeting HO-1 with macrophages may be a treatment choice for diabetes (Fig. 2A).

Other studies have confirmed that HO-1 obstructs the initiation of inflammation and ROS by directly regulating the activation of immune cells, including the antigen-presenting cells (APCs) and lymphocytes [41–43]. HO-1 was first demonstrated to block the maturation process of dendritic cells to exert a suppressive effect on inflammatory response in 2005 [44]. HO-1 expression was significantly reduced during dendritic cell maturation in vitro, which corresponds to the absence of HO-1 expression by mature dendritic cells in human tissues. Induction of HO-1 expression in dendritic cells can maintain their immature state, further preventing polarization of T cells towards to inflammatory phase (T_H_1 cells and T_H_17 cells) and promoting T_reg_ cell responses [45–47]. Related findings have also been found in the treatment of diabetes. Compared with uninduced mice, HO-1 expression induced in dendritic cells showed a lower incidence of T1DM and reduced the risk of insulitis (Fig. 2B). Induction of HO-1 expression in dendritic cells also prevented further hyperglycemia in recently diabetic NOD mice [19]. HO-1 regulated immune balance by reducing the expression of pro-inflammatory T_H_1 cytokines towards a beneficial T_H_2 pattern, restoring T_reg_ cell responses in diabetic mice and T2DM patients [48, 49]. These observations suggested that induction of HO-1 ameliorates detrimental inflammation in a variety of diseases, which also exhibited the preventive therapeutic approaches and management strategies of HO-1 expression for diabetes.

Another curative function of the HO-1 is to facilitate the conversion of heme into BV, Fe^2+^ and CO in equimolar quantities, thus promoting efficient antioxidant and anti-inflammatory effects [50]. The BV produced during these metabolic processes is rapidly transformed into BR through the action of BV reductase, which has a beneficial impact on numerous biological processes [51, 52]. HO-1 has been demonstrated to inhibit the inflammatory response via the simultaneous production of two anti-inflammatory molecules, CO and BR, as well as the removal of the pro-inflammatory factor heme [53]. The administration of hemin results in the boost of HO-1 expression and mitigates inflammatory responses in retinal ganglion cell injury in diabetic retinopathy, as well as diabetic wound healing, diabetic kidney function and [54–56]. CO can selectively impede the functioning of TNF-α, IL-1β and macrophage inflammatory proteins, which are pro-inflammatory cytokines, triggered by LPS [57, 58]. Meanwhile, CO actively promoted the production of the anti-inflammatory cytokine class IL-10 [59]. CO also suppressed the function of early growth response protein 1 (EGR-1), a crucial transcription factor in the inflammatory response initiated by macrophages [60]. Inflammation plays a pivotal role in exploring the pathophysiology of diabetic complications. The damage induced by hyperglycemia was generally regarded as results from a series of chain reactions triggered by inflammatory responses, leading to increased activity of the transcription factor NF-κB. This in turn enhances the activity of pro-inflammatory cytokines like TNF-α and leukocytes. Numerous studies have demonstrated that HO-1 has a significant effect on diabetic complications, with induction of HO-1 protein expression enhancing antioxidant defense and reducing inflammatory factors (Table 2). Notably, induction of HO-1 expression alleviated diabetic neuropathic pain and facilitated modulation of the diabetic nerve functions [61, 62]. The crucial role of HO-1 in safeguarding neurons against inflammation is also supported by related experiments with the Nrf2 inhibitor ML385 and si-HO-1 [63]. Therefore, HO-1, as a key factor in the inflammatory response, is expected to provide effective strategies for the prevention and treatment of diabetic complications and can provide novel ideas and drug targets for the prevention and treatment of clinical diseases.

## The role of HO-1 in programmed cell death in diabetes mellitus

Cell death is a fundamental physiological process in living organisms, which can be programmed or triggered by accident. Programmed cell death is an active and orderly way of death determined by genes. When host cells are attacked by exogenous and endogenous stimulating factors, different signaling pathways lead to lysis or non-lytic morphology [64]. Apoptosis is a widely studied mild non-lytic mode of cell death characterized by a decrease in cell volume and fragmentation of the nucleus and involved in many diseases including diabetes [65]. Conversely, lytic cell death is highly inflammatory, such as pyroptosis, necroptosis, and necrotic extracellular traps (NETosis) [66].

Pyroptosis, a pro-inflammatory programmed cell death triggered by activated Caspase-1/Caspase-11, which can lead to inflammatory cytokines IL-18/IL-1β release to activate pro-inflammatory immune cell mediators. NLRP3 is an inflammasome sensor protein that can perceive various DAMPs (danger-associated molecular patterns) and PAMPs (pathogen-associated molecular patterns). DAMPs including mitochondrial dysfunction, ionic flux confusion, and the active oxygen generation, which trigger pyroptosis and have been implicated in the kidney pathogenesis of many diseases including diabetes. The ASC as a small intermediary protein between Caspase-1 and most inflammasomes; the PYD domain of ASC interacts with the PYD of inflammasomes, and its caspase activation domain and CARD domain interact with the CARD of Caspase-1 to form the NLRP3 complex. After the inflammatory complex activation of NLRP3, Caspase-1 is markedly activated by the NLRP3 complex to form active Caspase-1. The precursors of IL-18 and IL-1β are cleaved into biologically active mature IL-18 and IL-1β by activated Caspase-1 and secreted through the cell membrane pyrototic pores. On the other hand, gasdermin D is cleaved into N-terminus and C-terminus by activated Caspase-1. N-terminal fragments promote the formation of cell membrane pyroptotic pores, which further leads to pyroptosis [67, 68]. Pyroptosis plays an important role in the progression of many diseases, including diabetes and its complications. Studies have shown that the protein level of HO-1 was downregulated and NLRP3/Caspase-1 was significantly activated, while HO-1 induction could reverse the activation of these pyroptosis-related proteins. This mechanism was validated by dapagliflozin (hypoglycemic agents) and atorvastatin (hypolipidemic agents) treatment in diabetic mice kidney and PA/high glucose-induced podocytes [69, 70]. Syringaresinol is a natural plant-derived polyphenolic compound, triptolide as a complex triepoxide diterpene natural product, similar protective effects have been observed in diabetic kidney-related studies [71, 72]. HO-1 was also found to be involved in a protective effect by inhibiting pyroptosis in diabetic cardiomyopathy [73].

**Fig. 3:**
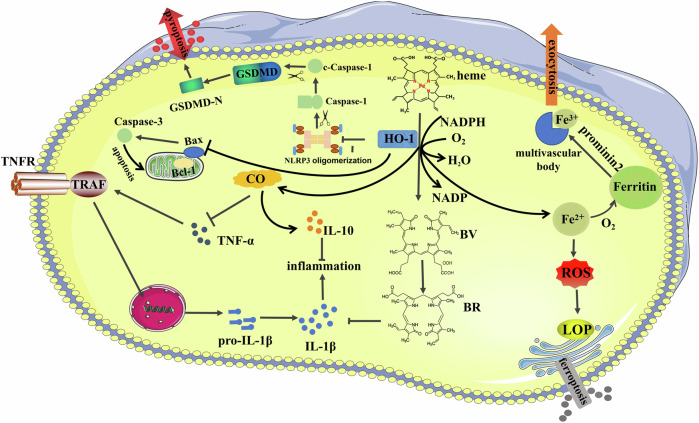
Enzymatic heme catabolism and regulation of HO-1 and its catalytic products in programmed cell death. Heme is catalyzed by HO-1 to form CO, Fe^2+^, and BV, and BV forms BR under the catalysis of biliverdin reductase. Both BV and BR have cellular antioxidant activities. Iron can act as a pro-oxidant or can trigger the synthesis of ferritin and play a role as a synergistic cytoprotective agent. CO can regulate a variety of cellular processes such as inflammation, apoptosis. In addition, HO-1 is also involved in the process of pyroptosis and ferroptosis.

Ferroptosis is an innovative mechanism of cell death that is mostly caused by the deposition of lipid peroxides and unevenness in the regulation of iron levels due to disruption of intracellular metabolic pathways. Ferroptosis is significantly different from the previously known apoptosis or autophagy in cell morphology, in which cell membrane rupture does not occur during ferroptosis. The mitochondrial membrane became denser and the outer membrane appeared ruptured, the structure of the cristae disappeared and the volume decreased. However, the volume of the nucleus does not change significantly, there is a lack of chromatin condensation but the chromosome structure does not disappear, and the rupture of the cell membrane does not occur in the process of ferroptosis. Ferroptosis is biochemically characterized by the generation of ROS and iron overloading. ROS and intracellular iron accumulation are important processes that trigger the mitogen-activated protein kinase (MAPK) pathway, decrease intracellular glutathione (GSH) depletion, and limit cystine absorption. Meanwhile, the activity of the cysteine-glutamate transporter protein system XC- activity is inhibited, further exacerbating the process. Glutathione peroxidase 4 (GPx4), is the only enzyme in cells that can reduce lipid peroxides to normal phospholipid molecules, its activity is crucial for maintaining normal cell function and ferroptosis [74]. Inhibition of cysteine-glutamate transporter protein system (e.g., Erastin) will deplete intracellular GSH, eventually resulting in the inactivation of GPx4 and the accumulation of lipid peroxidation, which can induce cell ferroptosis to a certain extent [75]. Furthermore, cellular antioxidant capability is also decreased by suppressed GPx4 expression (e.g., RSL3), which results in lipid peroxidation and ferroptosis [76]. The molecular regulation and pharmacological mechanisms of ferroptosis in disease and therapy are of great significance, and increasing evidence suggests that ferroptosis is associated with the pathogenesis of diabetes-related complications (Table 2). Quercetin is an important flavonoid with a variety of protective properties, it has been shown that quercetin inhibits ferroptosis in renal tubular epithelial cells by activating the HO-1 signaling pathway to regulate the expression level of antioxidant enzymes and by reducing intracellular ROS production and iron overloading [77]. However, another study showed the process of ferroptosis may exacerbate proteinuria, damaging renal tubules and promoting renal fibrosis via the HIF-1α/HO-1 pathway in diabetes models. The levels of HIF-1α and HO-1 were raised in diabetic kidney tissue exacerbated tubular iron accumulation, enhanced the lipid peroxidation response, and augmented the generation of ROS. Ferrostatin-1 significantly alleviates renal tubular injury and renal scar formation in db/db mice by preventing ferroptosis and reducing HIF-1α and HO-1 [78]. Numerous studies have shown that vitamin D receptor plays a positive role by inhibiting ferroptosis [79–81]. Paricasitol as a VDR agonist can inhibit ferroptosis by activating VDR/Nrf2/HO‐1 signaling pathway in DN, which effectively attenuates iron deposition in renal tubular epithelial cells and renal damage after diabetic injury [82]. At the same time, the elevated HO-1 expression helps to scavenge free radicals such as peroxynitrite, which is achieved through the overexpression of BV, thus ultimately inhibiting the process of lipid peroxidation [83]. In conclusion, the relationship between HO-1 and programmed cell death in diabetes deserves further investigation (Fig. 3).

## Role of HO-1 in regulating diabetic cardiomyopathy

Diabetic cardiovascular disease is the microvascular and macrovascular disease of the cardiovascular system caused by diabetes, which includes coronary heart disease, diabetic cardiovascular autonomic neuropathy, and diabetic cardiomyopathy [84]. Compared with non-diabetic patients, the incidence and mortality of cardiovascular disease in diabetic patients are higher [85]. The key risks of cardiovascular disease in diabetes are vascular endothelial dysfunction and impaired angiogenesis [86, 87]. Vascular dysfunction serves as the primary underlying cause of cardiovascular illness occurrence in patients experiencing anomalous glucose metabolism. The majority of diabetic fatalities arise from vascular impairments, including myocardial infarction and cerebrovascular afflictions. Insulin resistance, as well as the conditions of hyperinsulinemia, hyperglycemia, and elevated free fatty acids, play a substantial role in causing injury to cardiomyocytes, deteriorating their functionality, and leading to myocardial lipotoxicity in individuals with diabetes [88]. Diabetic cardiomyopathy increases mortality in diabetic patients by causing heart failure and is characterized by a multitude of interrelated systems that are influenced, including calcium homeostasis, renin-angiotensin system, protein kinase C signaling pathway, metabolism, mitochondria, fibrosis, and oxidative stress [89]. Interstitial fibrosis occurs after cardiomyocyte sclerosis, and collagen cross-links through late glycosylation products, ultimately leading to contractile dysfunction [90].

Induction of HO-1 expression is critical for preventing vascular dysfunction, endothelial cell death, and elevated ROS levels [91]. Moreover, research conducted in vitro has indicated the remarkable antioxidant properties of the bilirubin, which is derived from HO-1. Bilirubin has demonstrated the ability to enhance the protection of vascular endothelial cells, even when the concentration of this compound is in the nanomolar range. Bilirubin prohibits oxidative stress injury by directly scavenging reactive oxygen radicals and indirectly by restraining the activity of nitrogen compounds [92, 93]. HO-1 plays a significant function in vascular tissues by safeguarding endothelial cells from various apoptotic stimuli, thereby regulating endothelial cell cycle control, proliferation, vascular endothelial growth factor (VEGF) secretion, and angiogenesis [94, 95]. Furthermore, the increased expression of HO-1 in individuals suffering from heart failure serves to mitigate the adverse effects of pathological left ventricular remodeling, diminish myocardial hypertrophy, minimize oxidative stress, and abate inflammatory activation [96]. HO-1^–/–^ mice were used to prove that the deletion of HO-1 would aggravate myocardial injury in ischemia/reperfusion, especially related to diabetes. Compared with the HO-1^+/+^ mice (21.4 ± 1.8%), the myocardial infarct area significantly reached at 36.4 ± 20% in HO-1 deficient mice. Furthermore, the specific induction of HO-1 had a protective effect in diabetic mice suffering from myocardial ischemic injury [97]. Research in mice lacking HO-1 has revealed the overall function of HO-1 in restoring functional macrophages, maintaining iron balance within tissues, and increasing tolerance to oxidative stress conditions [98–100]. The protective effects of HO-1 induction in cardiac tissues can be further demonstrated by prolonging the survival time of allogeneic cardiac grafts, decreasing the mortality rate, and preserving the left ventricle [101, 102]. Generally, HO-1 levels remain low across diverse tissues except for the spleen, but diverse stimuli (like hydrogen peroxide, UV radiation, endotoxins, and hypoxia) can cause a strong inducible response for shielding cells from damaging oxidative and inflammatory agents [34]. Though limited, research data proposes activation of HO-1 as a prospective approach to amplify endothelial cell longevity with HO-1 byproducts CO and bilirubin, which could postpone the emergence of diverse cardiovascular complications related to diabetes. However, these studies are still in the preliminary stage, and the specific mechanism of action and effects need further research and validation [91, 103]. Therefore, we can expect more studies to reveal the specific role of HO-1 in diabetic cardiomyopathy in the future.

In addition to its direct effects on the cardiovascular system, HO-1 may also indirectly affect the occurrence and development of cardiovascular diseases in diabetes by regulating other biological processes. For example, HO-1 can affect insulin sensitivity and glucose metabolism, thereby improving glycemic control in diabetic patients [104]. Furthermore, HO-1 can affect biological mechanisms such as inflammatory response and oxidative stress, both of which significantly contribute to the onset of cardiovascular diseases precipitated by diabetes. Besides, HO-1 improved cardiovascular function by regulating cellular signaling pathways and promoting angiogenesis, etc (Table 2).

AMPK is an energy receptor that is activated when cells are low on energy and regulate multiple metabolic pathways through phosphorylation to maintain energy homeostasis. In diabetic cardiomyopathy, energy metabolism in cardiomyocytes may be impaired due to factors such as hyperglycemia and insulin resistance. Therefore, induction of HO-1 may activate AMPK pathway by enhancing the phosphorylation, thereby improving energy metabolism in cardiomyocytes [105, 106]. Autophagy is an intracellular degradation process that provides energy and nutrition by degrading damaged organelles and macromolecules [107]. In diabetic cardiomyopathy, the autophagy pathway may be inhibited, which leads to the accumulation of harmful substances in cardiomyocytes. LC3-II and Beclin-1 are key molecules in the autophagy pathway, induction of HO-1 activates the autophagy pathway by increasing the expression of LC3-II and Beclin-1, thereby removing harmful substances from cardiomyocytes [108]. Cardiomyocyte apoptosis and cardiac fibrosis were increased in the mouse model of diabetic cardiomyopathy, the protein expression of Nrf2 and HO-1 was reduced in left ventricular cardiomyocytes. After treatment with salidroside, Nrf2, and HO-1 were significantly restored, cardiac apoptosis, hypertrophy, and fibrosis were also improved [109]. Myricetin is a naturally occurring flavonol with a strong antioxidant effect. Studies have shown that myricetin significantly increased the activity of the Nrf2/HO-1 pathway to enhance the resistance to oxidative stress, demonstrated the reversal of glutathione peroxidase (GPx) and superoxide dismutase (SOD) activities, and reduced malondialdehyde (MDA) production [110]. High glucose-induced accumulation of persistent peroxidation in cardiomyocytes triggers ferroptosis and results in cell damage in H9C2 cells and New Zealand rabbits. Curcumin is a natural phenolic antioxidant, can increases Nrf2 transfer into the nucleus and promote HO-1 expression, reducing the excessive downregulation of GPx4 [111]. Besides, HO-1 was also involved in the protective effects of curcumin against pyroptosis, apoptosis, and oxidative stress in diabetic cardiomyopathy [73, 112].

**Fig. 4:**
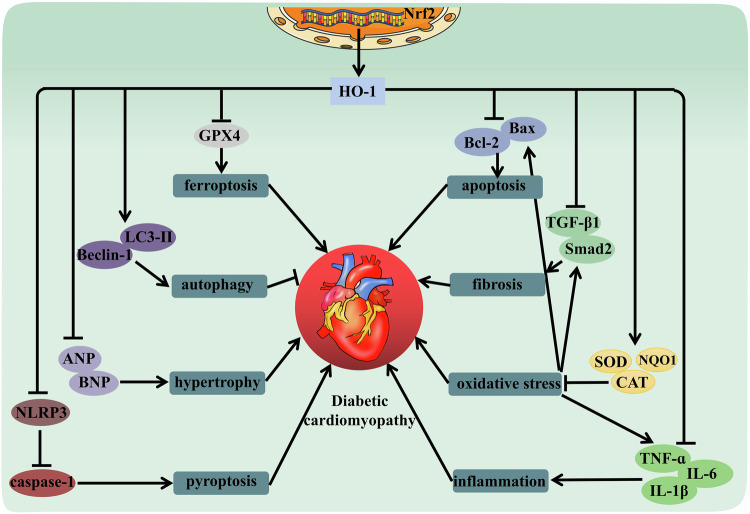
HO-1 may be involved in diabetic cardiomyopathy by regulating various signaling pathways. The increased expression of HO-1 can be reduced oxidative stress, inflammation, ferroptosis, pyroptosis, and apoptosis, improve myocardial hypertrophy and fibrosis. Notably, HO-1 plays a protective role by enhancing autophagy.

In summary, the utilization of HO-1 as a multifunctional molecule shows remarkable promise in improving cardiovascular dysfunction, especially in diabetes-related cardiovascular diseases (Fig. 4). Its superior properties in terms of shielding cardiovascular well-being stem from its potential as both an antioxidant and anti-inflammatory agent. Despite the immense potential of HO-1 in mitigating the manifestation of diabetes-related cardiovascular disorders, there exists a dearth of studies that explore its precise action mechanism and applicability. By reviewing deeper into the mechanism of HO-1 functions, we hope to devise innovative therapeutic measures that are aimed at preventing and curing diabetes-associated cardiovascular complications, ultimately enhancing the well-being and prospects of the patients concerned.

## HO-1 is involved in diabetic wound healing

Diabetes mellitus has a range of complications, among which diabetic foot ulcer (DFU) stands as a significant one. The diabetic environment often results in impaired wound healing processes for DFU, mainly attributed to excess oxidative stimulation, prolonged inflammation, dysfunction of immune cells, delayed reinnervation, and reduced angiogenesis at the wound site [113, 114]. The significance of neovascularization, inflammation, and apoptosis might imply that HO-1 plays a role in governing the process of wound healing [115].

Several studies have shown that hyperglycemia affects skin wound healing in diabetic rats [116, 117]. Induction of HO-1 reduced inflammatory cytokines, such as TNF-α and IL-6, increased antioxidants, and improved angiogenesis in wound tissues of diabetic rats, thereby wound healing was accelerated in the diabetic state [118]. Along with its angiogenic and cytoprotective enzyme functions, HO-1 performed crucial biological activities, making it highly significant in the wound healing process. Its enzymatic activity generates bioactive end products like CO, BV, and BR, which have a mediating effect on wound healing [119–121]. It was shown that increased HO-1-derived CO production has a vascular protective effect on T1DM, reduces endothelial cell fragmentation, and decreases ICAM-1, VCAM-1 expression, and Caspase-3 activity [122].

Diabetes patients exist in excessive oxidative stress and inflammation, these factors impair the response to skin damage [123]. HO-1 serves as a strong antioxidant that possesses anti-inflammatory as well as cell proliferation-promoting abilities. When the skin is damaged, the process of hemolysis releases hemoglobin (a pro-oxidant), which can further contribute to the induction of HO-1 [124]. Furthermore, HO-1 expression can be triggered by oxidative stress, inflammation, and hypoxia, and HO-1 and its products of enzymatic activity have the effect of reducing oxidative stress and pro-inflammatory factors. More importantly, they also promote cell viability, production of anti-inflammatory factors, as well as cell migration, proliferation, and angiogenesis, thus contributing to the healing process of diabetic wounds. A major factor contributing to chronic wound healing failure in diabetic patients is ROS overproduction and decreased antioxidant defenses [125]. The activation of HO-1 serves as a vital cellular defense mechanism against oxidative stress and possesses robust antioxidant properties. Hemin acts as a potent inductor of HO-1, which promotes the healing process through the reduction of oxidative stress as is evident by the diminished level of lipid peroxidation, and elevated levels of various antioxidant defense mechanisms, including GSH, SOD, GPx, and catalase [55, 120]. The presence of HO-1 significantly reduces the levels of inflammatory markers associated with diabetic wounds such as TNF-α, IL-1β, IL-6, iNOS, COX2, MCP-11, and MIP-1, while simultaneously boosting the levels of IL-10 [126].

HO-1 also plays an immunomodulatory effect in a variety of immune cells. In macrophages, increased HO-1 expression shows the ability to convert mobilized M1 macrophages into alternately activated M2 macrophages with anti-inflammatory effects, which is essential for the wound healing phase [127]. The process of wound healing requires the presence of angiogenesis. It is a commonly accepted fact that wounds in diabetic patients exhibit decreased levels of angiogenic factors, resulting in impairment of angiogenesis [128, 129]. HO-1 has the potential to stimulate the angiogenesis by elevating the expression of several pro-angiogenic factors including VEGF, translational growth factor-1 (TGF-1), and stromal-derived factor-1 (SDF-1) [130, 131]. As a methoxyindole secreted by the pineal gland, melatonin can increase the number of EPCs and enhance EPCS-mediated angiogenesis by induction HO-1 [132]. The anti-apoptotic properties of HO-1 have also been fully confirmed in several studies. Gynura divaricata (L.) DC. (GD), a traditional Chinese herbal medicine with hypoglycemic effects promotes angiogenesis and granulation tissue growth, which contribute to faster wound healing in diabetic SD rats. At the same time, GD improves HUVECs cells survival efficiency, reduces ROS generation and cell apoptosis, restores MMP, improves migration ability, and increases VEGF expression. These beneficial effects were abolished when inhibition was performed by using Nrf2-siRNA. It suggested that GD activates Nrf2 signaling pathway to increase HO-1 expression to regulate the expression of related proteins [133]. As a glucagon-like peptide-1 (GLP-1) receptor agonist used clinically to treat type 2 diabetes mellitus, liraglutide improves endothelial dysfunction caused by hyperglycemia, thereby preventing angiogenesis disorders in C57BLKs/J mice of wound model. Liraglutide also induction HO-1 expression by activating AMPK and promoting HIF-1α protein export from cytoplasm to nucleus, which significantly increases the expression of anti-apoptotic protein Bcl-2 and decreases the expression of pro-apoptotic Bax and Caspase-3 in HUVECs cells [134]. Turmeric-derived nanoparticles functionalized aerogel (TDNPs) can regulate the polarization balance between pro-inflammatory M1 and anti-inflammatory M2 in RAW 264.7 and restore macrophage-fibroblast communication network in L929 cells to ameliorate diabetes wound healing. Moreover, TDNPs have been verified to activate the Nrf2/HO-1 signaling pathway to promote the proliferation and migration of fibroblasts by improving their endogenous antioxidant capacity and reducing cell apoptosis [40]. Pyroptosis has also been confirmed to be involved in diabetic wounds, and cold atmospheric plasma can reduce major mediators NLRP3, Caspase-1, and IL-1β [58, 135]. However, there is no conclusive evidence to associate pyroptosis with HO-1 in diabetic wounds. Chronic hyperglycemia can induce circulating accumulation of lipid peroxidation products and impaired iron metabolic pathways, which results in the presence of a variety of free iron in plasma. Intracellular iron overload and accumulation of lipid peroxides are the characteristics of ferroptosis, so ferroptosis is considered as one of the potential mechanisms of delayed wound healing in diabetics [136, 137]. The mechanism of HO-1 in ferroptosis pathway of diabetic wounds has been rarely explored, and we believe that this is a work worthy of further investigation. We can excavate the value and significance of diabetic wound healing through a comprehensive and systematic study.

**Fig. 5:**
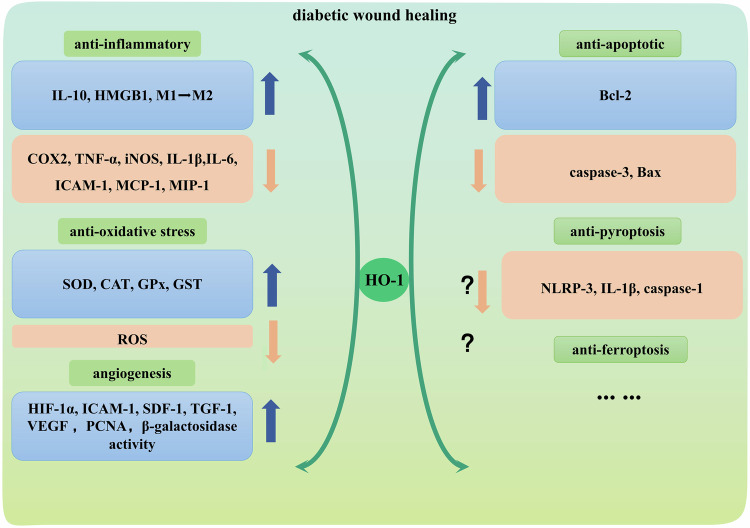
Beneficial role of HO-1 in promoting diabetic wound healing. Diabetic wounds have excessive oxidative stress and inflammation. Up-regulation of HO-1 will reduce oxidative stress and pro-inflammatory factors, and also promote cell viability, migration, proliferation, and angiogenesis, thus promoting diabetic wound healing.

Significant progress has been made in improving tissue healing and regeneration, but treatment options for untreated patients with chronic diabetes remain limited. Regulation of the complex wound healing process has proved to be a challenging task in diabetic patients due to impaired cellular function due to an excessive oxidative and inflammatory environment. HO-1 is considered as a promising therapeutic approach because of its crucial role as a marker in wound healing phases. Regulating HO-1 activity could be an effective strategy in treating diabetic foot ulcers, given its significance in every stage of wound healing. This highlights the potential to exploit HO-1 as a valuable target for therapeutic intervention in DFU cases (Fig. 5).

## Development and role of HO-1 in regulating diabetic nephropathy

Diabetic nephropathy (DN) is a widespread factor contributing to the morbidity and mortality in patients with T1DM and T2DM. End-stage diabetes is increasingly becoming one of the main causes of chronic renal failure, timely intervention of DN to prevent its development into end-stage renal disease has become a priority. DN is pathologically characterized by mesangial matrix thickening, progressive destruction of glomerular and tubulointerstitial, and loss of functioning glomeruli, ultimately leading to chronic renal failure [138, 139]. Various factors are involved in the progression of DN, such as disorder of oxygen metabolism and lipid metabolism, hyperglycemia, advanced glycosylation products, and inflammatory response [140, 141]. What makes sense is that persistent HO-1 stimulation diminishes hyperglycemia and enhances glucose metabolism while shielding renal tissues, partially or entirely, against hyperglycemic injuries. It is expected that HO-1’s antioxidant capabilities are accountable for this protection [142]. With multiple studies considering HO as a feasible target, the treatment of DN seems to have new prospects. There exist three distinct isoforms of HO referred to as HO-1, HO-2, and HO-3. HO-1 is renowned for its positive impacts including anti-apoptotic, antioxidant, anti-nitrosative, as well as anti-inflammatory effects against iNOS among three isoforms.

In various diseases, reduction of reactive oxygen species (ROS) accumulation by HO-1 induction. DM and its complications are significantly influenced by ROS. The role of ROS in the pathogenesis of DM, specifically in the development of DN, has been significantly highlighted [143]. When ROS accumulates excessively, it will cause oxidative stress damage to cells. ROS can cause oxidative damage to unsaturated fatty acids, proteins, DNA, and other important molecules in cells. In turn, these damaged cells are the targets of elimination by the immune system, which results in the production of endogenous injury-related molecular patterns and the release of cytokines. Inflammation is usually triggered by these dangerous molecular patterns and factors [144]. A variety of drugs have been reported to alleviate renal tissue damage by reducing ROS and inflammation, most of which is achieved through the Nrf2/HO-1 signaling pathway (Table 2). Shenkang injection is an active ingredient extracted from four medicinal plants: Radix et Rhizoma Salviae Miltiorrhizae, Radix Astragali, Flos Carthami, Radix et Rhizoma Rhei. Shenkang injection can improve the kidney function of diabetic SD rats by increasing the expression of antioxidant enzyme GPx4 and promoting the activation of antioxidant system. The molecular mechanism may be via the Keap1/Nrf2/HO-1 axis modulation [10]. Baicalin, a flavonoid, effectively increases the levels of GSH-PX, SOD, and catalase, inhibits the infiltration of inflammatory cells such as T-lymphocytes, T-helper cells, neutrophils, and macrophages, as well as the mRNA levels of pro-inflammatory cytokines (IL-1β, IL-6, MCP-1, and TNF-α) [145]. The relief of oxidative stress and inflammation in kidney of C57BLKs/J mice is due to the activation of Nrf2/HO-1 and MAPK signaling pathways. Notoginsenoside R1 promoted HO-1 expressions to reduce oxidative stress-induced apoptosis and kidney fibrosis in HK-2 cells and C57BL/6J mice [146]. As described above, AMPK are sensors and protectors of cellular energy needs. Echinochrome A, a natural bioproduct extracted from sea urchin, protects mitochondrial function against oxidative stress damage by activating AMPK phosphorylation and regulating the AMPKα/Nrf2/HO-1 pathway [147].

Diabetic renal fibrosis is stimulated by multiple factors such as inflammation, which forms a large amount of improper proliferation and excessive deposition of collagen fibers. In the process of development, there will be renal tubular atrophy, glomerular sclerosis, and microvascular sparse, forming a vicious circle and finally, the kidney completely loses its organ function [148]. HO-1 plays an important role in diabetic kidney fibrosis (Table 2). By regulating the activity or expression of HO-1, it may provide a new strategy for the treatment of diabetic renal fibrosis. Astaxanthin reduces the expression of FN, ICAM-1, and TGF-β1 induced by HG to reduce ECM deposition, and significantly promotes the nuclear translocation and transcriptional activity of Nrf2, and induction the expression of HO-1 [149, 150]. In another in vitro and in vivo study, Nrf2/HO-1 was also demonstrated to play an important inhibitory role in the pro-fibrotic pathway activated by TGF-β1, which was verified by oligo-fucoidan in NRK-52E cells and kidney tissues of C57BL/6 mice [151].

Current studies have unraveled that programmed cell death has a notable effect on DN progression. Apoptosis is a widely explored mechanism of programmed cell death, and apoptosis of renal tissue is a key feature of diabetic kidney injury [152]. HO-1 as an important regulator of oxidative stress and inflammation, also plays an important role in diabetic kidney apoptosis. HO-1 was involved in the protective effect of telmisartan against DN, and the mRNA levels regulation of Nrf2 and HO-1 indicated that telmisartan had a regulatory effect on apoptosis besides anti-inflammation and anti-oxidation [153]. In addition to the excellent therapeutic effect on diabetic cardiomyopathy, sinapic acid also ameliorated the expression of apoptosis-related proteins in STZ-induced Wistar rats’ diabetic kidneys via Nrf2/HO-1 mediated pathways [154]. Pyroptosis is an important programmed cell death pattern that can be activated by DAMPs and PAMPs. Dapagliflozin can decrease podocyte pyroptosis mediated through the miR-155-5p/HO-1/NLRP3 signaling pathway, and induction of HO-1 can promote the inhibitory effect of dapagliflozin on pyroptosis [69]. Similar protective effects on podocyte pyroptosis mediated by HO-1 were proved by multiple drugs, including atorvastatin, triptolide. A study based on Nrf2-KO (knockout of Nrf2) diabetic mice showed that syringaresinol inhibits pyroptosis caused by NLRP3/Caspase-1 in DN by activating the Nrf2/HO-1 signaling pathway [72]. Ferroptosis is a programmed cell death with iron-dependent accumulation of lipid peroxides, which has recently been identified in animal models of DN [155]. After ferroptosis was inhibited by ferrostatin-1 treatment in C57BLKs/J mice, urinary albumin to creatinine ratio was decreased, kidney tubular injury and kidney fibrosis were improved, HIF-1α and HO-1 expression were suppressed [78]. In HK-2 cells and C57BL/KsJ mice, the kidney jury restoration after ferroptosis inhibition via the Nrf2/HO-1 signaling pathway has been sufficiently confirmed in the research on the therapeutic mechanism of quercetin, vitamin D receptor, umbelliferone, etc [77, 82, 156]. In SV40-MES 13 cells, HMGB1 (high-mobility group box-1) has been recognized as a valuable factor of ferroptosis based on the Nrf2/HO-1 signaling pathway, proposing that HMGB1 is a new target for the treatment of DN [157]. This indicated that HO-1 exhibits excellent benefits in ferroptosis of DN since it is an important downstream target affected by HGBM1.

**Fig. 6:**
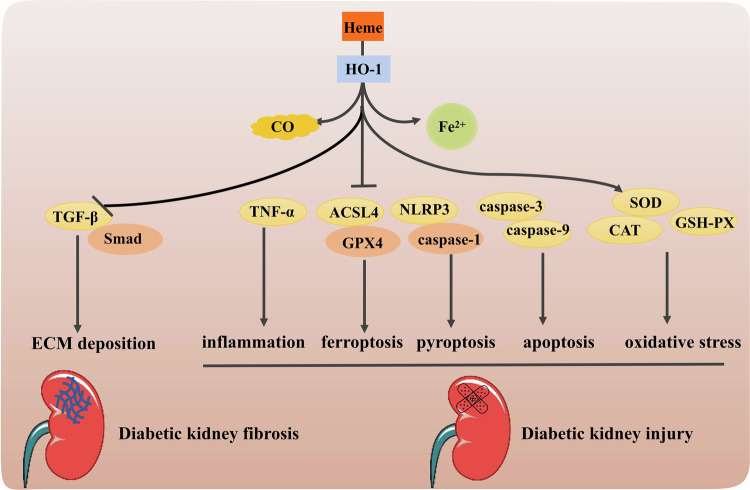
Schematic diagram of the signaling pathway of HO-1 in the protection of diabetic nephropathy. The increased expression of HO-1 can reduce ECM deposition to improves kidney fibrosis, diminish inflammation, oxidative stress, ferroptosis, pyroptosis and apoptosis to alleviate diabetic kidney injury.

Thus, HO-1 plays significant part in regulating the progression and application of DN (Fig. 6). Through a deeper knowledge of the biological function and regulatory mechanism of HO-1, it can provide groundbreaking ideas and strategies for the therapy of diabetic nephropathy. Nonetheless, the utilization of HO-1 target in the treatment of diabetic nephropathy is still in the examination stage and requires further clinical trials for its efficacy and safety. We hope that in the future, more researchers will focus on more basic research on HO-1 in DN, including its expression regulation, mechanism of action, and interaction with other molecules. Based on these researches, clinical trials targeting HO-1 should be carried out to evaluate its application in the treatment of DN, so as to provide evidence for clinical application.

## Protective role of HO-1 in other complications of diabetes

Diabetic retinopathy (DR) is a series of retinal microvascular diseases caused by DM, which has a great impact on vision and even blindness in the late stage. An increasing number of clinical and laboratory studies have revealed the pathophysiological changes of DR [158]. The protective effect of HO-1 in diabetic retinopathy is not only reflected in the inhibition of inflammation and oxidative stress but also promoted microglia polarization from the M1 state (pro-inflammatory) towards the M2 state (anti-inflammatory) [159]. And HO-1 related to programmed cell death such as apoptosis and ferroptosis in DR was also revealed by hydroxysafflor yellow A(a single chalcone glycoside), maresin-1 (pro-resolving lipid mediator), amygdalin (natural cyanogenic glycoside) [160, 161]. Multiple studies have shown that diabetes is associated with cognitive impairment, in which HO-1 as an important antioxidant enzyme, plays an important role in protecting against apoptosis, inflammation, and oxidative stress in diabetic cognitive dysfunction [162–164]. HO-1 is also involved in the protection of mitochondrial dysfunction and oxidative stress in diabetic Alzheimer's [165, 166]. What’s more, HO-1 can halt ferroptosis related to diabetes, such as diabetes with sepsis, diabetes-induced liver injury, diabetic encephalopathy, and diabetic islet transplantation [167–170]. Through in-depth study of the mechanism of HO-1, it is expected to provide new strategies and methods for the treatment of diabetes complications.

## Conclusion

DM and its associated complications are significant maladies that affect human health, which pose a serious threat to the wellness of the global population. The persistent intricacies of diabetes stem from various metabolic pathway disorders, ultimately resulting in substantial health risks and danger of death associated with the condition. The HO-1 plays a crucial role in process of triggering and advancing diabetes and its associated complications. In this investigation, the function and correlation that HO-1 with diabetes and its complications have been comprehensively examined. Studies have shown that HO-1 may be involved in the development of diabetic complications like diabetic kidney, diabetic cardiovascular disease, and diabetic wound healing. Most studies have focused on HO-1 affecting diabetic complications through its antioxidant, anti-apoptotic, and anti-inflammatory effects, but HO-1 has also shown a role in programmed cell death that cannot be ignored. Further research is essential to thoroughly investigate HO-1’s regulatory mechanisms, its interaction with other transcription factors, and its functions across varying kinds and phases of diabetes. Further, there is a need to concentrate on examining the function and correlation of HO-1 in diabetes and its complications, which will pave the way for innovative approaches to avoid and treat diabetes. However, HO-1 induction also brings negative effects. Induction of HO-1 causes abnormal accumulation of ROS due to Fe^2+^ accumulation, inducing ferroptosis. It also causes cellular CO toxicity and bilirubin encephalopathy. These findings suggest that HO-1 is not merely protective in disease and requires a comprehensive understanding.

In conclusion, exploring the new molecular mechanism of HO-1 in the treatment of diabetes can provide new methods and ideas for the diagnosis and treatment of diabetes and related diseases, and offer the molecular theoretical foundation for the development of new hypoglycemic drugs.
